# *N. elongata* Produces Type IV Pili That Mediate Interspecies Gene Transfer with *N. gonorrhoeae*

**DOI:** 10.1371/journal.pone.0021373

**Published:** 2011-06-22

**Authors:** Dustin L. Higashi, Nicolas Biais, Nathan J. Weyand, Al Agellon, Jennifer L. Sisko, Lewis M. Brown, Magdalene So

**Affiliations:** 1 Department of Immunobiology and the BIO5 Institute, University of Arizona, Tucson, Arizona, United States of America; 2 University Spectroscopy and Imaging Facilities, University of Arizona, Tucson, Arizona, United States of America; 3 Department of Biological Sciences, Columbia University, New York, New York, United States of America; Monash University, Australia

## Abstract

The genus *Neisseria* contains at least eight commensal and two pathogenic species. According to the *Neisseria* phylogenetic tree, commensals are basal to the pathogens. *N. elongata*, which is at the opposite end of the tree from *N. gonorrhoeae*, has been observed to be fimbriated, and these fimbriae are correlated with genetic competence in this organism. We tested the hypothesis that the fimbriae of *N. elongata* are Type IV pili (Tfp), and that Tfp functions in genetic competence. We provide evidence that the *N. elongata* fimbriae are indeed Tfp. Tfp, as well as the DNA Uptake Sequence (DUS), greatly enhance *N. elongata* DNA transformation. Tfp allows *N. elongata* to make intimate contact with *N. gonorrhoeae* and to mediate the transfer of antibiotic resistance markers between these two species. We conclude that Tfp functional for genetic competence is a trait of a commensal member of the *Neisseria* genus. Our findings provide a mechanism for the horizontal gene transfer that has been observed among *Neisseria* species.

## Introduction

Acquisition of novel genetic traits can increase fitness of an organism and augment its chances of survival in changing environments. Transfer of genetic material between bacteria occurs mainly through conjugation, transduction and transformation (uptake of exogenous DNA) [1], [2], [3], [4], [5]. Interspecies gene transfer is less well understood. Bacteria belonging to the genus *Neisseria* provide a good opportunity to examine the role of the Type IV pilus in interspecies horizontal gene transfer.

The Type IV pilus (Tfp) mediates DNA uptake and transformation in many bacteria, including the pathogenic *Neisseria*, *N. gonorrhoeae* and *N. meningitidis* [6], [7], [8]. *N. gonorrhoeae* Tfp are peritrichous fibers measuring ∼6 nm in diameter and several microns in length. These fibers are composed primarily of pilin subunits arranged in a structured helix [9], [10]. Among the approximately 20 genes involved in the biogenesis of Tfp, four are absolutely essential for its assembly: *pilE*, encoding pilin; *pilD*, encoding the prepilin peptidase; *pilF*, encoding an ATPase that assembles processed pilins into the Tfp fiber; and *pilQ*, encoding subunits of the outermembrane pore through which the growing fiber extends [11].

Over eight species of commensal *Neisseria* commonly colonize human mucosal epithelia [12], [13], [14]. Despite their prevalence, little is known about the biology of these organisms. A recent genomics study revealed that commensals are basal members of the *Neisseria* genus; the two pathogens, *N. gonorrhoeae* and *N. meningitidis*, evolved from a common ancestor of the commensals, with *N. lactamica* being their closest relative [15]. All *Neisseria* species harbor genes for Tfp biogenesis [15], [16] as well as multiple copies of the 10 base pair DNA Uptake Sequence (DUS; GCCGTCTGAA). Both have been shown to function in genetic competence in the pathogenic *Neisseria* [17], [18]. A number of commensals are naturally competent for genetic transformation [19], [20]. *N. elongata*, a basal member of the *Neisseria* genus, is observed to be fimbriated, and the fimbriae are correlated with genetic competence [19], [21].

We tested the hypothesis that commensal *Neisseria* express Tfp, and that the structure functions in genetic competence. We focused our studies on *N. elongata* as it represents a basal member of the human *Neisseria* genus [15]. We report that *N. elongata* produces Tfp. Genetic competence in *N. elongata* is greatly enhanced by Tfp and the presence of DUS. Tfp also allows *N. elongata* to make intimate contact with *N. gonorrhoeae* and mediates the bi-directional transfer of antibiotic resistance markers between them. Our findings provide one mechanism for the widespread horizontal gene transfer that has been observed among *Neisseria* species [15], [19], [22].

## Results

### *N. elongata* cells are competent for DNA transformation to rifampicin resistance

*N. elongata* can be genetically transformed to streptomycin resistance [19], [21]. We first determined the frequency at which *N. elongata* subspecies *glycolytica* acquires rifampicin resistance when incubated with DNA from a variant of the same strain that had spontaneously developed resistance to rifampicin (Rif; *N. elongata* rif^R^). When incubated with rif^R^ DNA, *N. elongata* acquired rifampicin resistance at a >3,400-fold higher frequency than when incubated with medium or with DNA in the presence of DNaseI (Table 1). Similar results were obtained with *N. elongata* subspecies *elongata* (data not shown). This study confirms that our strain of *N. elongata* is genetically transformable and establishes the frequency of rif^R^ transformation.

**Table 1:** Transformation of *N. elongata* with rif^R^ chromosomal DNA.

Strain	DNA	Transformation Frequency^a^ (×10^−5^)
*N. elongata* (wt)	*N. elongata* rif^R^	7.12±1.54
*N. elongata* (wt)	No DNA	<0.00204±0.00192
*N. elongata* (wt)	*N. elongata* rif^R^ + DNaseI	<0.000108±0.000006
*N. elongataΔpilE::Km*	*N. elongata* rif^R^	0.0210±0.0197
*N. elongataΔpilE::Km*	No DNA	<0.000050±0.000008

a Transformation frequency is expressed as the number of rif^R^ bacteria/total CFUs. Values are averaged from three independent experiments ± SEM. “<” indicates the transformation frequency was below the limit of detection of the assay.

### Key Tfp biogenesis genes are transcribed in *N. elongata*

**Figure 1:**
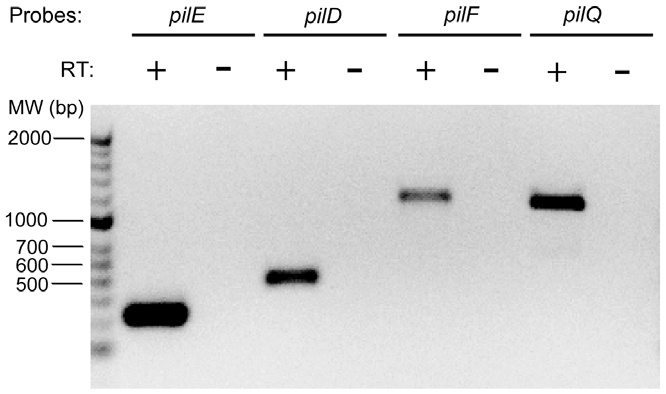
Tfp biogenesis genes in *N. elongata* are transcribed. Migration of PCR amplicons generated from *N. elongata* cDNA using primers specific for *pilE*, *pilD*, *pilF*, and *pilQ*. (+) and (−) indicate the presence or absence of reverse transcriptase (RT) in the cDNA reaction.

Tfp biogenesis and functionality are dependent on the expression of multiple genes. The above result led us to determine whether Tfp biogenesis genes in *N. elongata* are transcriptionally active. We focused on four genes essential for Tfp assembly: *pilE*, *pilD*, *pilF*, and *pilQ* [11]. To detect their transcripts, *N. elongata* RNA was reverse transcribed into cDNA and the products were probed by PCR using primers specific for each gene. Amplicons of the sizes expected for all four genes were obtained (Figure 1). Amplicons could not be generated when reverse transcriptase was omitted from the cDNA reactions. Sequencing of these amplicons confirmed the identity of these genes (data not shown). Thus, *pilE*, *pilD*, *pilF*, and *pilQ* are transcribed in *N. elongata*.

### *N. elongata* produces Tfp

**Figure 2:**
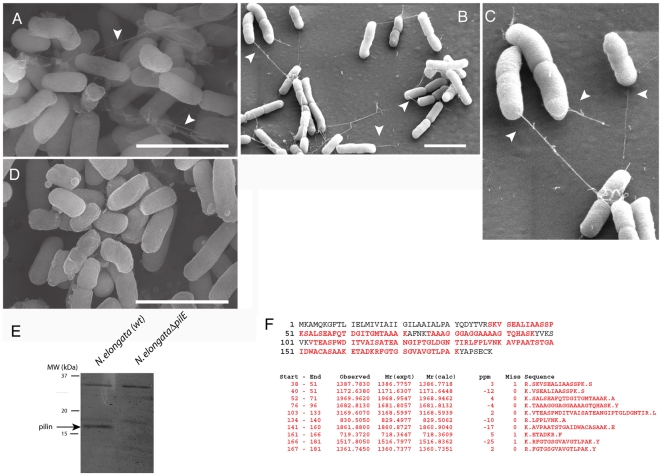
*N. elongata* produces Type IV pili. Scanning Electron Microscopy (SEM) of Wt *N. elongata*, (A), (B) and (C) and *N. elongata*Δ*pilE* (D). (C) is an enlarged image of the upper left hand section in (B). Arrowheads indicate Tfp-like fibers. Scale bars: 2 µm. (E) SDS PAGE of fibers isolated from wt *N. elongata* and *N. elongata*Δ*pilE* using a protocol for isolating *N. gonorrhoeae* Tfp. Arrow indicates the 17 kDa protein. (F) Top panel: Amino acid sequence deduced from the *N. elongata pilE* gene. Bottom panel: Sequences of peptides from the *N. elongata* 17 kD protein determined by tryptic digestion and MALDI-TOF mass spectroscopy. Deduced amino acid sequences that match the peptide sequences are in red.

**Figure 3:**
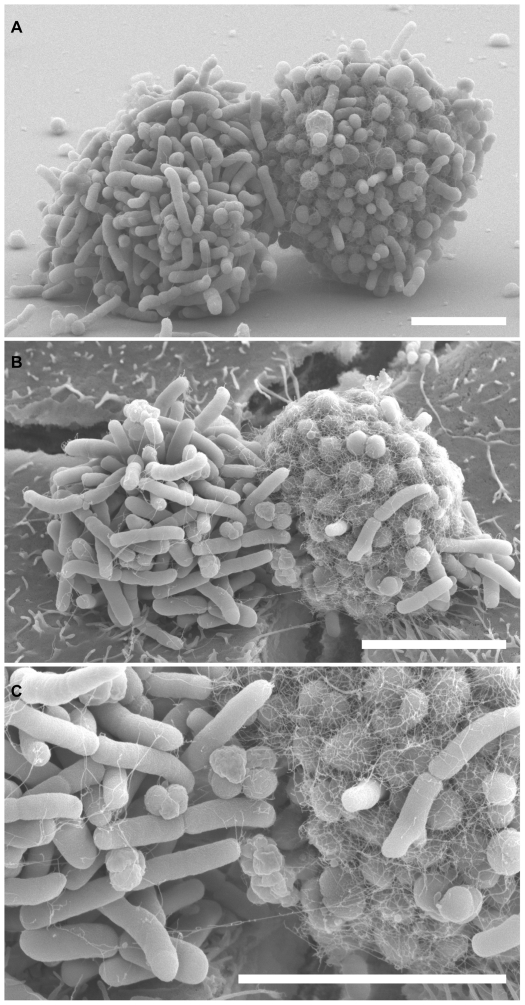
*N. elongata* and *N. gonorrhoeae* make intimate contact with each other on abiotic surfaces and human epithelial cells. SEM of *N. elongata* (rods) and *N. gonorrhoeae* (cocci) co-cultured 3 hours on (A) glass coverslips, and (B) human epithelial cells. (C) A higher magnification image of a region in (B). Scale bars: 5 µm.

Fimbriae have been observed in *N. elongata* cells [21]. We examined *N. elongata* for evidence of Tfp by Scanning Electron Microscopy (SEM) (Figure 2A). *N. elongata* cells are slender rods [19] that typically measure 0.5 µm by ∼2 microns. Tfp-like fibers were present on *N. elongata*, though they were not as abundant as Tfp on *N. gonorrhoeae* [23] (see also Figure 3). Consistent with a previous report [21], these Tfp-like fibers appear to originate from one end of the long axis of the cell (Figure 2B and 2C). Quantitative experimentation will be required to determine the exact location of the *N. elongata* fibers. It should be noted that Tfp fibers in other gram negative rods are polar [5], [24], [25]; the polar nature of these fibers may be linked to the motility and detachment behavior of these organisms [26].

Fibers from *N. elongata* were isolated using a protocol developed for obtaining *N. gonorrhoeae* Tfp [27]. The predominant protein in these preparations had an electrophoretic mass of ∼17 kD (Figure 2E), the approximate size of *N. gonorrhoeae* pilin. Data from tryptic digestion and MALDI/TOF spectroscopy of the 17 kD protein agreed with the theoretical mass and deduced amino acid sequence of *N. elongata pilE* [15] (Figure 2F). PilE is therefore the major constituent of *N. elongata* fibers.

### The *N. elongata pilE* null mutant does not produce Tfp and is significantly less transformable

To obtain conclusive proof that *N. elongata* fibers are Tfp, we determined whether a *pilE* null mutant produced fibers. The *pilE* gene in *N. elongata* was replaced with a kanamycin (Km) resistance cassette (see Materials and Methods). This mutant, *N. elongataΔpilE*::Km, grew at the same rate as the wt parent strain (data not shown). Mutant cells were devoid of fibers, as judged by SEM (Figure 2D). Neither fibers nor the 17 kD protein could be purified from *N. elongataΔpilE*::Km cells (Figure 2E). Taken together, these findings demonstrate that the *N. elongata* fibers are Tfp.

To test the DNA transformation function of *N. elongata* Tfp, we incubated wt and *N. elongataΔpilE*::Km with DNA purified from the rif^R^ strain. *N. elongataΔpilE*::Km transformed to rifampicin resistance at a ∼340-fold lower frequency than the wt parent (Table 1). The low frequency of DNA transformation of *N. elongataΔpilE*::Km is consistent with that observed for non-piliated *N. gonorrhoeae* [28]. Tfp therefore plays an important role in *N. elongata* DNA transformation.

### *N. elongata* DNA transformation is enhanced by a DNA uptake sequence

DNA uptake and transformation by pathogenic *Neisseria* is significantly enhanced by the canonical DNA Uptake Sequence (DUS; GCCGTCTGAA) [17], [18], [29]. We determined whether transformation of *N. elongata* is also enhanced by this DUS (Table 2). Cells incubated with a DNA fragment encoding a segment of *rpoB* from *N. elongata* rif^R^ and DUS (*rpoB^rifR^* + DUS) transformed to rifampicin resistance at an ∼9000-fold higher frequency than cells incubated with medium or with the amplicon in the presence of DNaseI.

**Table 2:** Role of DUS in DNA transformation by wt *N. elongata.*

DNA	Transformation Frequency^a^ (×10^−5^)
none	<0.000191±0.000011
*rpoB^rifR^*	0.0104±0.0041
*rpoB^rifR^* + DUS	1.71±0.35
*rpoB^rifR^* + DUS1	0.211±0.083
*rpoB^rifR^* + DUS + DNaseI	<0.000174±0.000029

a Transformation frequency is expressed as the number of rifampicin resistant bacteria/total CFUs. Values represent the average from three independent experiments ± SEM. “<” indicates the transformation frequency was below the limit of detection of the assay. “none”: medium only.

DUS1, a variant DUS with a thymine at the second position (GtCGTCTGAA), also functions in DNA transformation of the pathogenic *Neisseria*, but at a lower frequency than the canonical sequence [30]. *N. elongata* was transformed by the *rpoB^rifR^* + DUS1 amplicon, but at an 8-fold lower frequency than the *rpoB^rifR^* + DUS amplicon (Table 2).

The *rpoB^rifR^* amplicon alone also transformed *N. elongata* to rifampicin resistance, but at a low frequency compared to the *rpoB^rifR^* + DUS amplicon (Table 2). One possible explanation for this event is that the amplicon entered cells through a DUS- and DUS1-independent mechanism. DUS-independent transformation is known to occur at low levels in *N. gonorrhoeae* [31], [32]. Regardless, our results demonstrate that transformation of *N. elongata* is most efficient when the DNA contains the canonical DUS. At least one DUS variant, DUS1, also functions in transformation at a low frequency.

### *N. elongata* physically interact with *N. gonorrhoeae* on synthetic surfaces and epithelial cells

Commensal and pathogenic *Neisseria* inhabit similar niches on mucosal epithelia, raising the possibility of interspecies interactions and transfer of genetic material. We first determined whether *N. elongata* and *N. gonorrhoeae* could physically interact with each other. After 3 hours of co-culture, *N. elongata* and *N. gonorrhoeae* formed microcolonies that were largely monospecific (Figure 3A). However, many *N. elongata* and *N. gonorrhoeae* microcolonies were observed abutted to each other. Moreover, a number of *N. elongata* cells were observed within *N. gonorrhoeae* microcolonies and *vice versa*. *N. gonorrhoeae* Tfp, which formed a network over microcolonies, also attached to neighboring *N. elongata* cells (Figure 3A). This intimate association of commensal and pathogen also occurred in mixed infections of human epithelial cells (Figure 3B and 3C). *N. elongata* and *N. gonorrhoeae* microcolonies frequently interacted with each other on glass surfaces and epithelial cells: 80% of randomly selected *N. elongata* microcolonies were physically associated with a *N. gonorrhoeae* microcolony on glass and 81% on cells (±3% and ±5%, respectively; n ≥50 for each type of surface; see Methods). Very few *N. elongata* and *N. gonorrhoeae*Δ*pilE* mutants adhered to glass surfaces or epithelial cells when cultured alone or together. The few Δ*pilE* mutants of each species that adhered did not aggregate into microcolonies and did not interact with each other when co-cultured (data not shown). Together, these data indicate that Tfp promotes physical interactions between *N. elongata and N. gonorrhoeae*.

### *N. elongata and N. gonorrhoeae* engage in bi-directional horizontal gene transfer

The above findings led us to determine whether *N. gonorrhoeae* could transfer genetic material to *N. elongata*. *N. gonorrhoeae* rif^R^ and *N. elongata* sensitive to rifampicin (rif^S^) cells were co-cultured, and transfer of rif^R^ from pathogen to commensal was measured (Table 3). *N. elongata* acquired rif^R^ at a >21-fold higher frequency when co-cultured with *N. gonorrhoeae* rif^R^ than when co-cultured with *N. gonorrhoeae* rif^S^. Acquisition of resistance is enhanced by Tfp, as *N. elongata*Δ*pilE::Km* acquired rif^R^ from *N. gonorrhoeae* rif^R^ at a ∼38-fold lower frequency than the wt *N. elongata* parent.

**Table 3:** Transfer of antibiotic resistance marker between *N. elongata* and *N. gonorrhoeae*.

Donor	Recipient	Transformation Frequency^a^ (×10^−7^)
*N. gonorrhoeae* rif^S^	*N. elongata* rif^S^	0.0657±0.0532
*N. gonorrhoeae* rif^R^	*N. elongata* rif^S^	1.39±0.71
*N. gonorrhoeae* rif^R^	*N. elongata*Δ*pilE::Km* rif^S^	0.0366±0.0162
*N. gonorrhoeae* rif^R^	none	NG*
*N. elongata* rif^S^	*N. gonorrhoeae* rif^S^	0.351±0.182
*N. elongata* rif^R^	*N. gonorrhoeae* rif^S^	5.60±3.95
*N. elongata* rif^R^	*N. gonorrhoeae*Δ*pilE* rif^S^	0.118±0.067
*N. elongata* rif^R^	none	NG**

a Number of rif^R^ recipient bacteria/total number of recipient bacteria (see Methods for differential selection of each species.).

*No growth of *N. gonorrhoeae* on LB Lennox agar.

**No growth of *N. elongata* on GCB/VCN agar. Values represent the average from three independent experiments ± SEM.

Finally, we determined whether commensal could transfer genetic material to pathogen, using the same approach. *N. gonorrhoeae* acquired rif^R^ at a >15-fold higher frequency when co-cultured with *N. elongata* rif^R^ than when co-cultured with *N. elongata* rif^S^ (Table 3). As expected, transfer of rif^R^ is enhanced by Tfp, as *N. gonorrhoeae*Δ*pilE* acquired rif^R^ from *N. elongata* rif^R^ at a 47-fold lower frequency than the wt *N. gonorrhoeae* parent. The lower transformation frequencies observed in co-culture assays compared to assays with purified DNA have been reported for *N. gonorrhoeae* [33]. These results demonstrate that *N. elongata* and *N. gonorrhoeae* can engage in bi-directional transfer of genetic information during co-culture.

## Discussion

According to the *Neisseria* phylogenetic tree, commensals species are basal to the two pathogenic species, *N. gonorrhoeae* and *N. meningitidis* [15]. We presented evidence that the most basal *Neisseria* species, *N. elongata*, expresses Tfp that is functional for genetic transformation. These results, together with the observation that all commensal species harbor Tfp biogenesis genes and DUS elements [15], suggest that Tfp is an ancestral trait of the *Neisseria* genus, and that this trait has been inherited by the two pathogens as they evolved from a common ancestor.

*N. elongata* is efficiently transformed by DNA containing the canonical DUS, GCCGTCTGAA. It is also transformed at a low frequency by DNA containing the DUS1 variant GtCGTCTGAA. This result is consistent with the lower transformation efficiency of DUS1 for pathogenic *Neisseria* [30]. The DUS is the most highly repeated sequence in *Neisseria* genomes, occurring >2,000 times in most species [15]. *N. elongata* has 2,142 copies of the DUS and 117 copies of DUS1 [15]. Interestingly, *N. sicca* and *N. mucosa*, which form a distinct clade in the *Neisseria* phylogenetic tree, have >3,400 copies of DUS1 but many fewer copies of DUS. Whether the relative abundance of these two DNA uptake sequences may influence genetic exchange between the *N. sicca/N. mucosa* clade and other *Neisseria* species remains to be determined.

Commensal *Neisseria* are part of the normal flora of mucosal epithelia. Pathogenic *Neisseria* can also persist asymptomatically for periods of time in similar niches [12], [34], [35]. Indeed, *N. elongata* and *N. gonorrhoeae* are known to inhabit the same mucosal environments [35], [36], [37], [38], [39]. This situation creates opportunities for interspecies interactions. Within 3 hours of co-culture, planktonic *N. elongata* and *N. gonorrhoeae* cells form largely monospecific microcolonies that physically contact microcolonies of the other species; a few *N. elongata* cells are observed within *N. gonorrhoeae* microcolonies and *vice versa*. How these interactions develop over time remains to be determined. Nevertheless, our results show that *N. elongata* and *N. gonorrhoeae*, representing distantly related species in the *Neisseria* phylogenetic tree, have the ability to interact with each other.

Tfp also plays an important role in the bi-directional transfer of genetic information between *N. elongata* and *N. gonorrhoeae*. Previous studies uncovered evidence of widespread horizontal gene transfer among *Neisseria* species, including antibiotic resistance alleles and genes known or proposed to play a role in virulence [15], [21], [40]. Our findings provide a mechanism for this observation.

## Materials and Methods

### Bacterial strains and epithelial cell line

*N. elongata* subspecies *glycolytica* (ATCC # 29315) and *N. gonorrhoeae* strain MS11 (P+, Opa-nonexpressing) were used [15], [20], [41]. Strains spontaneously resistant to Rifampicin were isolated by plating on agar containing Rifampicin (15 or 50 mg/L). The rifampicin resistant mutant of *N. elongata* (*N. elongata* rif^R^) contains a T to C transition that resulted in a change of serine to proline at residue 540 of RpoB. The rifampicin resistant mutant of MS11 (*N. gonorrhoeae* rif^R^) contains a C to T transition that resulted in a change from histidine to tyrosine at residue 553 of RpoB. To construct *N. elongata*Δ*pilE*::Km, a Kanamycin (Km) cassette flanked by 250 base pairs of DNA immediately upstream and downstream of the *pilE* ORF was transformed into wt *N. elongata*. Km resistant transformants were screened by PCR and sequencing. One mutant, *N. elongata*Δ*pilE* was selected for phenotypic studies. The MS11Δ*pilE* mutant is deleted in both pilin expression sites (Δ*pilE*1, Δ*pilE*2) [42], [43]. Bacteria were grown on GCB agar (Difco) with Kellogg's supplements at 37°C, 5% CO_2_. Infections were carried out on the 16HBE14o^−^ human bronchial epithelial cell line, which supports *Neisseria* infection [44], [45]. 16HBE14o^−^ cells were maintained in Eagle's Minimal Essential Medium (MEM, Invitrogen, San Diego, CA) with 10% heat-inactivated fetal bovine serum (FBS, Gibco) at 37°C and 5% CO_2_ and grown in tissue culture flasks pre-coated with LHC (Laboratory of Human Carcinogenesis) basal medium (Invitrogen) supplemented with 0.01 mg/ml human fibronectin (BD Laboratories, New York City, NY), 0.029 mg/ml bovine collagen (BD Laboratories), and 0.1 mg/ml BSA (Invitrogen).

### RNA extraction, cDNA, and PCR, and sequence analysis

Bacterial RNA was extracted from *N. elongata* using the RNAeasy kit (QIAGEN) per manufacturer's instructions. DNA was removed using DNA-free (Ambion) and samples were quantified by spectrophotometry (NanoDrop, Thermo Scientific). Complementary DNA was generated using iScript cDNA Synthesis Kit (BioRad) per manufacturer's protocol. Oligonucleotides complementary to *pilE* (pilE.f.85, 5′CCGGCTTACCAAGACTACACT3′; pilE.r.497, 5′GCCAGAACCAGTACCGAAAC3′), *pilD* (pilD.f.203, 5′TAACTAAACCGGCATCACGA3′; pilD.r.780, 5′TAGCTAAGCTGGGACCGAAT3′), *pilF* (pilF.f.105, 5′AATGTTGTTTGCTGACGGAA3′; pilF.r.1244, 5′ACGAGACAATGTTGCAGGAG3′), *pilQ* (pilQ.f.547, 5′CAAGCTCAGCGTAATTTGGA3′; pilQ.r.1651, 5′TCAATTATCGCTTCTTTGCG3′) were purchased from Sigma-Aldrich. PCR was performed using GoTaq Green Master Mix (Promega) using manufacturer's recommendations for annealing temperatures for each primer pair. PCR products were sequenced on a 3730XL DNA Analyzer (Applied Biosystems). Sequence Alignments were done using BLAST (National Center for Biotechnology Information).

### Electron Microscopy, Microcolony Quantitation and Proteomics

For co-culture studies, *N. elongata* and *N. gonorrhoeae* were suspended as single cells in GCB containing Kellogg's supplements, each at a density of 5×10^7^ CFUs/ml. For co-culture on epithelial cells, 16HBE14o^−^ human bronchial epithelial [44], [45] cells were grown to 90% confluence on glass coverslips then co-infected for 3 hours with *N. elongata* and *N. gonorrhoeae*, each at an MOI of 50. For co-culture on abiotic surfaces, two mls of each bacterial suspension were inoculated onto a glass coverslip and incubated for 3 hours. For SEM studies of *N. elongata* Tfp, bacteria were suspended as described above and incubated for 3 hours on glass (for polarity studies) or spotted on 0.2 µm polycarbonate membrane filters (Whatman). Samples were washed in PBS, fixed successively in PBS containing glutaraldehyde (2.5% wt/vol) and OsO_4_ (1% wt/vol), and stained with uranyl acetate (2% wt/vol). Samples were washed in PBS and dehydrated by successive immersions in ethanol at the following concentrations: 15%, 30%, 50%, 70%, 80%, 90%, 95% (vol/vol, in water), and 100%. Samples were critical point dried and sputter coated with platinum, and imaged using the Hitachi S-4800 Field-Emission Scanning Electron Microscope. For quantitation of physical interaction between *N. elongata* and *N. gonorrhoeae* microcolonies, SEM images of microcolonies (see above) of co-cultures on glass (n≥50) and epithelial cells (n≥50) were selected for further analysis. In each case, *N. elongata* microcolonies were identified, then scored for the presence of a *N. gonorrhoeae* microcolony in physical contact. The number of *N. elongata* microcolonies physically contacting a *N. gonorrhoeae* microcolony is then divided by the total number of *N. elongata* microcolonies counted. The averages of three independent experiments and Standard Error of the Mean were calculated. Tfp preparations from *N. elongata* were isolated as described [10], [27]. Briefly, *N. elongata* grown for 16 hours on GCB plates were resuspended in 50 mM CHES (2-(Cyclohexylamino)ethanesulfonic acid from Sigma-Aldrich) pH 9.5. The suspension was vortexed for 2 minutes and the bacteria bodies were spun down at 18,000× g for 10 minutes. The supernatant was then collected and spun down at 100,000× g for 1.5 hours. The pellet was resuspended in 50 mM CHES. Preparations were separated by SDS PAGE; the region of the gel containing the 17 kDa protein was excised and subjected to in-gel trypsin digestion. Digests were analyzed by peptide mass fingerprinting on a Voyager DE-Pro mass spectrometer (Applied Biosystems) in reflectron mode, and with internal calibration on trypsin autolysis products. Peak lists generated by Mascot Wizard (Ver. 1.2.0.0) were submitted to a Mascot database server (Ver. 2.3) (Matrix Science Ltd.), and searched against the NCBI non-redundant database of 03/26/10 with 10,688,764 sequences and 3,647,636,407 residues. The search, using the following settings: maximum 1 missed cleavage, no modifications, and no taxonomic restriction, yielded matches for 10 mass values out of 67 submitted with a protein score of 126 for gi accession number 291308345 with 71% sequence coverage with 11 ppm RMS error (expectation = 2.7e-06). The highest-ranking non-homologous hit had a Mascot score of 61 (expectation = 8.5).

### Co-culture Transformation Assay

Donor and recipient bacteria were each adjusted to a concentration of 5×10^7^ CFU/mL in a total volume of 2 ml, in GCB medium with Kellogg's supplements and 5 mM MgS0_4_. The cultures were incubated together in 50 mm dishes at 37°C, 5% CO_2_ for 8 hours. To select for *N. gonorrhoeae*, cultures were plated on GCB agar with Kellogg's supplements and Vancomycin, Colistin and Nystatin (VCN) +/− Rifampicin (15 mg/mL). To select for *N. elongata*, cultures were plated on LB Lennox agar +/− Rifampicin (15 mg/mL). These selective media allowed the quantitation of rif^R^ bacteria of each species without background from the donor species. Transformation frequency is defined as the number of rif^R^ recipient bacteria divided by the total number of recipient bacteria.

### PCR amplification of rpoB rif^R^ gene segment and chromosomal DNA preparation

A segment of the *rpoB* gene (position 1492 to 2183) was amplified from *N. elongata* rif^R^ (described above) using oligonucleotide primers (Sigma-Aldrich) complementary to *rpoB*, with or without DUS sequences. The primer are: L1492 (5′CGTGTVGAACGTGC3′), R2183 (5′CCTTACCATCGGTTTTTCAG3′), R2183DUS (5′CCTTTTCAGACGGCACCATCGGTTTTTCAG3′), and R2183DUS1 (5′CCTTTTCAGACGACACCATCGGTTTTTCAG3′). DUS sequences are underlined. Amplification products were purified using QIAquick PCR Purification Kit (QIAGEN, Valencia, CA). Products were quantitated by spectrophotometry (NanoDrop, Thermo Scientific). For chromosomal DNA preparations, bacteria from one agar plate were resuspended in 0.5 ml of GC Lysis Buffer containing 1% SDS followed by RNAse A treatment (Qiagen RNAse). 0.9 ml of phenol/chloroform/isoamyl alcohol (at a 25∶24∶1 ratio) was added to the lysate and the solution was vortexed for 1 minute. DNA was extracted from the mixture using Phase Lock Gel system (5 Prime) per manufacturer's instructions. DNA was precipitated by the addition of 1 ml isopropanol, followed by 5 minutes centrifugation at 16,000× g. Isopropanol was removed and the DNA pellet air dried. The DNA pellet was dissolved in 0.3 ml of TE (10 mM Tris-HCl; 1 mM EDTA, pH 8.0), and reprecipitated by the addition of 150 µl 7.5 M NH_4_OAc and 1 ml of 100% EtOH. The DNA was pelleted by centrifugation for 10 minutes at 16,000× g, and the pellet was washed with ice cold 70% EtOH (vol/vol). The DNA was dried and resuspended in water, and the concentration determined by spectrophotometry as described above.

### DNA transformation experiments

Transformation assays were performed as described [46], [47]. Briefly, *N. elongata* or *N. elongata*Δ*pilE* were grown on supplemented GCB agar for 16 hours. A DNA mixture was made by the addition of chromosomal (2.0 µg) or PCR-generated DNA (0.5 µg) to 200 µl of pre-warmed GCB medium supplemented with 5 mM MgSO_4_. Bacteria were suspended in GCB liquid medium supplemented with 5 mM MgSO_4_, to an OD_600_ of ∼2. 20 µl of this bacterial suspension was added to the DNA mixture and incubated for 20 minutes at 37°C. The transformation mix was then added to 2 ml of supplemented GCB medium in a 50 mm tissue culture dish and incubated at 37°C, 5% CO_2_ for 5 hours. Bacteria were pelleted by centrifugation at 6500× g for 5 minutes. The pellets were suspended in transformation medium and serial dilutions were plated on GCB agar containing Rifampicin (15 mg/L) to enumerate antibiotic resistant bacteria, and on GCB plates without antibiotics to determine total CFUs.
